# Reliable and Cost-Effective QuEChERS–UHPLC–MS/MS Method for the Simultaneous Analysis of Ten Mycotoxins in Dendrobium Officinale: A Study on Food-Medicine Homology Quality Control

**DOI:** 10.1155/jamc/8995925

**Published:** 2025-09-23

**Authors:** Zejun Wang, Qingcheng Wang, Fei Xu, Linhua Wang, Jingjing Liang, Qing Shen, Shunyuan Guo

**Affiliations:** ^1^Laboratory of Medicine-Food Homology Innovation and Achievement Transformation, Linping Hospital of Integrated Traditional Chinese and Western Medicine, Hangzhou 311110, Zhejiang, China; ^2^Innovation Laboratory for Traceability and Authentication of Dao-di Traditional Chinese Medicine, Hangzhou Linping Hospital of Traditional Chinese Medicine, Linping 311106, Zhejiang, China; ^3^Food Inspection Institute, Zhejiang Provincial Institute for Food and Drug Control, Hangzhou 310052, China; ^4^Panvascular Diseases Research Center, The Quzhou Affiliated Hospital of Wenzhou Medical University, Quzhou People's Hospital, Quzhou 324000, China; ^5^Laboratory of Food Nutrition and Clinical Research, Institute of Seafood, Zhejiang Gongshang University, Hangzhou 310012, China; ^6^Department of Neurology, Center for Rehabilitation Medicine, Hangzhou Medical College, Zhejiang Provincial People's Hospital (Affiliated People's Hospital), Hangzhou 314408, China

**Keywords:** dendrobium officinale, matrix effect, mycotoxins, QuEChERS, sample purification, UHPLC–MS/MS

## Abstract

This study developed a method for simultaneously detecting 10 mycotoxins in Dendrobium officinale using the QuEChERS technique combined with ultra-high-performance liquid chromatography–tandem mass spectrometry (UHPLC–MS/MS). The method was optimized for sample purification, pretreatment, chromatographic conditions, and mass spectrometric settings. It effectively addressed the matrix effects from impurities like pigments and cellulose. The validation of the method showed good linearity (*R*^2^ > 0.990), with limits of detection (LODs) ranging from 0.23 to 8.61 μg kg^−1^ and limits of quantification (LOQs) from 0.77 to 28.7 μg kg^−1^. Average recoveries for the 10 mycotoxins ranged from 77.9% to 98.5%, with relative standard deviations (RSD) between 2.26% and 8.28%. The method demonstrated high accuracy, precision, and suitability for large-scale screening of mycotoxins in Dendrobium officinale. When applied to 84 samples, the contamination rate was 2.38%, with the main contaminants being AFB1, ZEN, and AFG1. This method provides a reliable, cost-effective approach for detecting mycotoxin contamination in traditional Chinese medicine.

## 1. Introduction

Dendrobium officinale, a perennial epiphytic herb belonging to the Orchidaceae family and the Dendrobium genus, primarily utilizes its stems as the medicinal part [1]. As one of the traditional Chinese medicines that can be used both as food and medicine, the medicinal history of Dendrobium officinale spans over a millennium and is widely distributed across regions, such as Asia and Europe [2]. This herb contains various bioactive components, including polysaccharides, alkaloids, flavonoids, and stilbenoids [3]. Modern pharmacological studies have demonstrated its anti-inflammatory, antioxidant, and immune-enhancing properties [4]. Clinically, Dendrobium officinale also exhibits effects on balancing glucose and lipid metabolism, improving insulin resistance, promoting insulin secretion, and enhancing pancreatic cell function and has a potential role in preventing and treating complications of diabetes [5].

Dendrobium officinale is susceptible to fungal contamination at multiple stages, including cultivation, collection, processing, storage, and transportation [6]. Fungi can proliferate within the herb, potentially degrading its precious active components and generating various mycotoxins, which can significantly impact the overall quality and efficacy of Dendrobium officinale [7]. Although Dendrobium officinale is extensively used in the food and traditional Chinese medicine industries, research on mycotoxin residues is far less in-depth and comprehensive compared to the food sector. Current studies on mycotoxins are relatively limited, mainly focusing on aflatoxins, while other highly toxic and frequently occurring toxins such as ochratoxin, fumonisins, and others receive significantly less attention and lack a systematic research framework [8, 9]. To effectively control mycotoxin contamination, some regions and countries have implemented mandatory regulations on the content of mycotoxins in medicinal herbs, setting strict limit standards.

The detection methods for mycotoxins mainly include ultra-high-performance liquid chromatography–tandem mass spectrometry (UHPLC-MS/MS), liquid chromatography, and enzyme-linked immunosorbent assay (ELISA), which are typically used to analyze individual or single-class mycotoxins [10–12]. Among these, UHPLC–MS/MS has gained extensive application due to its high selectivity and sensitivity in detecting multiple mycotoxins. However, the accuracy and sensitivity of this method are easily influenced by matrix effects (MEs), necessitating effective pretreatment steps to remove large molecular impurities and other interfering factors from the matrix [13, 14]. For herb samples, which mainly contain sugars, pigments, and cellulose, common pretreatment methods include liquid–liquid extraction (LLE), solid-phase extraction (SPE), and the QuEChERS (Quick, Easy, Cheap, Effective, Rugged, and Safe) method [15–17]. Compared to LLE and SPE, the QuEChERS method is faster, simpler, safer, and more widely applicable, with high extraction and purification efficiency, making it more suitable for the detection of large quantities of samples. Therefore, it holds significant potential in mycotoxin detection.

This study presents a novel approach by optimizing the QuEChERS–UPLC–MS/MS method specifically for Dendrobium officinale, a traditional Chinese medicinal plant that has received limited attention regarding mycotoxin contamination. In contrast to previous studies that primarily focused on a few mycotoxins, our research addresses a broader spectrum of contaminants by simultaneously detecting 10 mycotoxins. This method is simple and convenient to operate, highly sensitive and accurate, providing technical support for solving the problem of mycotoxin residue detection in traditional Chinese medicines and ensuring the quality of these medicinal herbs.

## 2. Materials and Methods

### 2.1. Chemicals and Reagents

Ten mycotoxin standards, aflatoxin B_1_ (AFB1), aflatoxin B_2_ (AFB2), aflatoxin G_1_ (AFG1), aflatoxin G_2_ (AFG2), deoxynivalenol (DON), ochratoxin A, zearalenone (ZEN), fumonisin B_1_ (FB1), fumonisin B_2_ (FB2), and T-2 toxin, were all provided by the Zhejiang Institute for Food and Drug Control, with purities ≥ 97%. This selection was based on two key factors: these 10 mycotoxins are frequently found in various agricultural products, making them pertinent to consumer safety, and each of these mycotoxins has documented adverse health effects, including carcinogenic properties, necessitating monitoring to ensure the safety of medicinal products. Methanol (MeOH) and acetonitrile (ACN), HPLC grade, were purchased from Fisher Scientific (MA, USA); formic acid (FA) and ammonium formate (AF), MS grade, were obtained from Sigma-Aldrich (Heidelberg, Germany); anhydrous magnesium sulfate (MgSO_4_) and sodium chloride (NaCl), analytical grade, were from Aladdin Biochemical Technology Co., Ltd. (Shanghai, China); nylon syringe filters (0.22 μm) were from Jinteng Experimental Equipment Co., Ltd. (Tianjin, China); and QuEChERS sorbents (MgSO_4_, PSA, and C_18_) were from Anpu Experimental Technology Co., Ltd. (Shanghai, China).

### 2.2. Sample Preparation

Accurately, we weigh 5 g of a homogeneous sample (± 0.01 g) into a 50-mL centrifuge tube, add 10 mL of water, vortex to mix, and soak for 10 min. Then, incorporate 2 g of sodium chloride and 15 mL of a mixture of ACN, water, and FA in the ratio of 80:20:0.1 (v/v/v). Vortex thoroughly for 2 min and shake for 15 min to ensure complete extraction of mycotoxins. Following this, centrifuge the mixture at 6000 g for 3 min. Carefully transfer the supernatant to another centrifuge tube. To ensure maximum recovery and purity, repeat the extraction process by adding an additional 10 mL of 0.1% FA in ACN to the remaining sample residues in the original tube. After vortexing and centrifugation, combine the supernatants from both extractions to achieve a final extract volume of approximately 25 mL. Next, take 2 mL of this combined extract and add 150 mg of MgSO_4_ and 50 mg of C_18_ sorbent. Vortex for 2 min followed by centrifugation at 6000 g for another 3 min. This supernatant is then collected and dried using nitrogen blowing at 40°C until nearly dry. The residue is dissolved in 2 mL of a reconstituted solution of ACN–water–FA (80:20:0.1, v/v/v) and filtered through a 0.22-μm membrane for further analysis.

### 2.3. UHPLC–MS/MS

A Waters Acquity BEH C_18_ chromatographic column (100 mm × 2.1 mm, 2.6 μm) is employed, with a column temperature maintained at 35°C and an automatic injector temperature at 10°C. Water with 0.1% FA is designated as mobile phase A, while water (0.1% FA)–MeOH (0.1% FA) serves as mobile phase B. For chromatographic performance comparison, peak shape score (PSS) quantifies the symmetry and sharpness of the peak by the ratio of the peak's maximum height to the width at half height; relative response intensity (RRI) is calculated as the ratio of the peak area of the target compound to that of the standard, assessing the response intensity of the target compound under different conditions.

The flow rate is set at 0.3 mL min^−1^, with gradient elution performed according to the following programs: 5% B to 40% B from 0 to 8 min, 40% B to 100% B from 8 to 10 min, 100% B from 10 to 12 min, 100% B to 5% B from 12 to 13 min, and 5% B from 13 to 15 min. An injection volume of 5 μL is used. The QTRAP 5500 triple quadrupole mass spectrometer (Sciex, USA) is utilized, equipped with an electrospray ionization (ESI) source operating in multiple reaction monitoring (MRM) mode for simultaneous detection of positive and negative ions. A nebulizer voltage of 5500 V and an ion transfer temperature of 500°C are established, with declustering voltage and collision energy detailed in Table 1.

### 2.4. ME

The ME, considered acceptable within ± 20%, is calculated using the formula: ME (%) = [(A2 − A1)/A1] × 100, where A1 is the average area of the mycotoxin standard in solvent at a specific concentration and A2 is the average area of the mycotoxin standard in blank spice extract at the same concentration. A result greater than 0 indicates a matrix enhancement effect, while a result less than 0 demonstrates a matrix suppression effect. Matrix solutions from a blank MeOH reagent and negative Dendrobium officinale samples after pretreatment are prepared, from which mixed standard solutions at a concentration of 10 μg L^−1^ for 10 mycotoxins are formulated. The signal peak response intensities of these mycotoxins are measured to assess the ME.

### 2.5. Method Validation

The method was validated in terms of linearity, sensitivity, recovery, and precision. The mixed standard stock solution of 10 mycotoxins was diluted using blank matrix extract to obtain a series of matrix standard solutions with different mass concentrations. A matrix-matched standard curve for each mycotoxin was established with the concentration (*x*, μg kg^−1^) on the *x*-axis and peak area (*y*) on the *y*-axis. The limit of detection (LOD) was determined as 3 times the signal-to-noise ratio (S/N), and the limit of quantification (LOQ) was determined as 10 times the S/N. Samples were processed according to the method described in Section 2.2, and spiking experiments were conducted by adding the mixed standard working solutions of 10 mycotoxins at low, medium, and high (μg·L^−1^) levels to the blank matrix of Dendrobium officinale. The recovery rates for each mycotoxin were calculated based on the ratio of the measured value to the theoretical value. The relative standard deviation (RSD) was calculated from the results of five parallel tests.

### 2.6. Data Processing

Data collection, qualitative, and quantitative analysis are carried out via the Analyst 1.6 workstation system. Subsequently, data analysis and graphical plotting are executed using the WPS Office 2022 software.

## 3. Results and Discussion

### 3.1. Evaluation of Extraction Solvents

**Figure 1:**
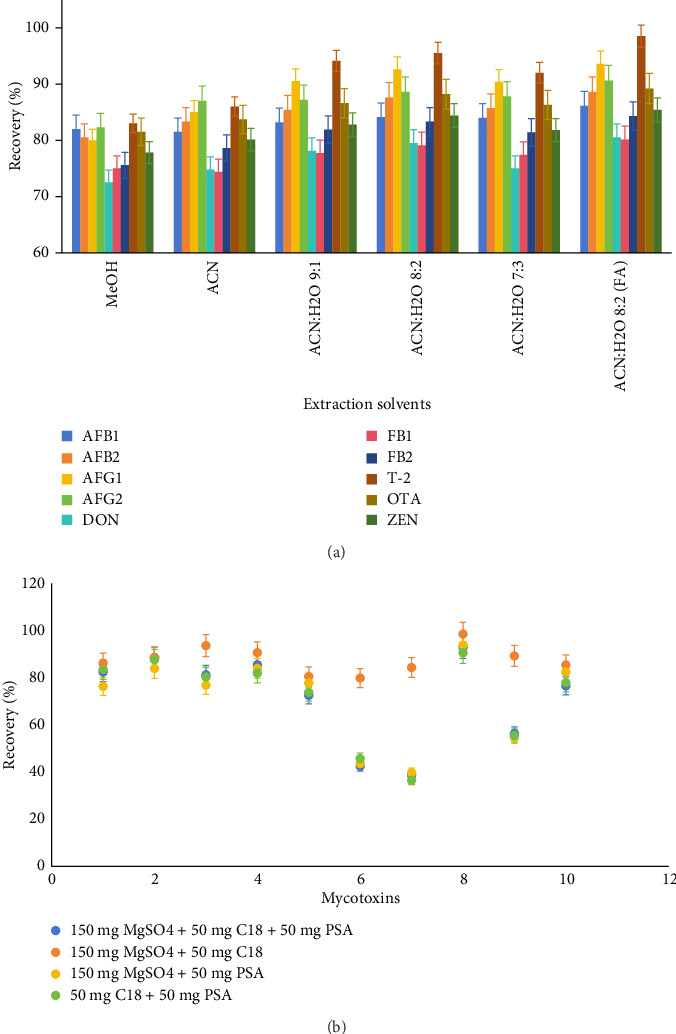
(a) Effect of different QuEChERS purification sorbents, Group A: 150 mg MgSO_4_ + 50 mg C_18_ + 50 mg PSA; Group B: 150 mg MgSO_4_ + 50 mg C_18_; Group C: 150 mg MgSO_4_ + 50 mg PSA; Group D: 50 mg C_18_ + 50 mg PSA, on the extraction of mycotoxins in Dendrobium officinale; (b) effect of different extraction solvents, including MeOH, ACN, ACN:H2O (9:1, v/v), ACN:H2O (8:2, v/v), and ACN: H2O (7:3, v/v) on the extraction of mycotoxins in Dendrobium officinale.

Extraction is a critical step in the QuEChERS technique, and its effectiveness directly determines the accuracy and practicality of the analytical results. MeOH and ACN are commonly used extraction solvents. The study found that for the 10 mycotoxins, there was no significant difference (*p* > 0.05) in the recovery rates of AFB2, OTA, FB1, and FB2 between MeOH and ACN, by using a two-tailed paired *t*-test. In contrast, the recovery rates of the remaining six mycotoxins exhibited significant differences. This is because MeOH and ACN are two commonly used universal extraction solvents, with ACN offering better selectivity than MeOH, avoiding the extraction of more fats, pigments, and other impurities [18]. ACN also has a higher extraction efficiency and better compatibility with LC–MS/MS. Therefore, ACN was chosen as the extraction solvent in this study. Additionally, among the mycotoxins tested in this study, fumonisins and ochratoxins have carboxyl groups and strong hydrophilicity. Using a high-organic phase system could result in partial loss of their recovery rates, necessitating the use of extraction solvents with water addition. However, a high proportion of water in the extraction solvent could dissolve a large amount of impurities from the matrix, reducing method sensitivity and consequently leading to a significant drop in the recovery rates of some toxins. Moreover, toxins like DON, ZEN, OTA, FB1, and FB2 are sensitive to acid, and lowering the pH of the extraction solvent can enhance stability and increase extraction efficiency [19]. Therefore, to ensure the extraction effect of various mycotoxins, auxiliary reagents need to be added to the extraction solvent to enhance the combined extraction effect. As shown in Figure 1(a), when FA and water were added to the extraction solvent, the resulting ACN–H_2_O–FA mixed solution (80:20:0.1, v/v/v) served as the extraction agent, effectively extracting the 10 mycotoxins. Each mycotoxin exhibited good peak shape and high intensity, making it suitable for qualitative and quantitative analysis.

### 3.2. Selection of Purification Sorbents

Due to the presence of a significant amount of impurities such as pigments, organic acids, tannins, and cellulose in the Dendrobium officinale samples, which are prone to contaminate the chromatographic and mass spectrometry systems and interfere with the detection of target substances, it is necessary to purify the Dendrobium officinale samples to reduce the ME [20]. Common purification materials include MgSO_4_, which has a good dehydration effect; C_18_, which effectively removes nonpolar impurities like fats and lipids; PSA, which is mainly used to remove carbohydrates, phenols, fats, and polar pigments; and graphite carbon black (GCB), which has a good adsorption effect on impurities with planar molecular structures such as sterols and chlorophyll [21]. Existing methods, such as those utilizing immunoaffinity columns, often experience limitations due to high costs and the need for extensive sample manipulations. These traditional methods primarily target specific classes of mycotoxins and may fail to effectively purify complex samples that contain a mixture of contaminants. For instance, studies incorporating SPE or LLE have shown varying recovery rates influenced by MEs, especially in herbal samples where plant compounds can interfere with the detection [22]. Preliminary studies have reported that some mycotoxins, such as ST, AFB1, and AFB2, have planar or partially planar molecular structures and are easily adsorbed by GCB, affecting their recovery rates [23]. Therefore, GCB was not used in this experimental sample processing. The effects of different combinations and proportions of purification materials on the recovery rates of mycotoxins in Dendrobium officinale were investigated, Group A: 150 mg MgSO_4_ + 50 mg C_18_ + 50 mg PSA; Group B: 150 mg MgSO_4_ + 50 mg C_18_; Group C: 150 mg MgSO_4_ + 50 mg PSA; Group D: 50 mg C_18_ + 50 mg PSA. These four combinations were chosen based on their proven abilities to effectively remove a variety of impurities and enhance the recovery of target mycotoxins. The results (Figure 1(b)) showed that the purification sorbents containing PSA had a strong adsorption effect on FBT and OTA, resulting in their recovery rates in Groups A, C, and D being only 36.5%–56.3%. When Group B was used, the recovery rates of the 10 mycotoxins were optimal, ranging from 79.8% to 98.5%. Therefore, 150 mg MgSO_4_ and 50 mg C_18_ were selected as the QuEChERS purification sorbents for purification and impurity removal of the extract.

### 3.3. Optimization of Chromatographic Conditions

**Figure 2:**
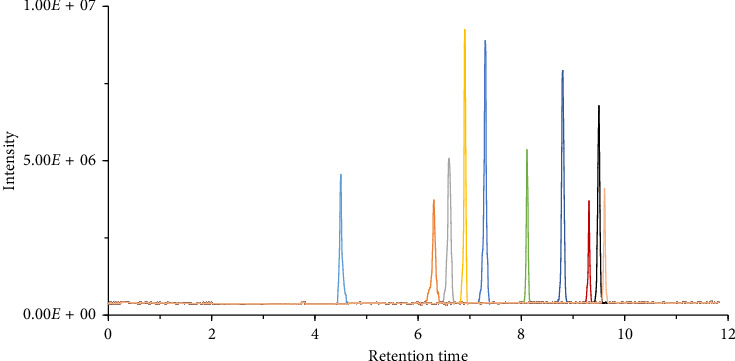
The chromatogram of the 10 mycotoxins using QuEChERS and UHPLC–MS/MS method under the optimized chromatographic conditions and mass spectrometric conditions.

Different types of chromatographic columns were evaluated, including Phenomenex Kinetex C_18_ column (150 mm × 2.1 mm, 2.6 μm), Agilent Zorbax SB-C_18_ column (150 mm × 2.1 mm, 3.5 μm), and Waters Acquity BEH C_18_ column (150 mm × 2.1 mm, 3 μm). In our experimental conditions, the Waters Acquity BEH C18 column quickly achieved effective separation of the target compounds, exhibiting excellent chromatographic peak shapes and clarity. It is important to note that this does not imply that the other two columns are unsuitable for mycotoxin analyses; rather, under the specific conditions tested, the Waters Acquity BEH C18 column exhibited superior performance. Therefore, this column was selected for the analysis in this study. The effectiveness of water–MeOH, water–ACN, water (0.1% FA)–MeOH (0.1% FA), and water (0.1% FA)–ACN (0.1% FA) as mobile phases for the separation of target compounds was investigated. The results indicated that the water (0.1% FA)–MeOH (0.1% FA) provided the best separation effect with minimal solvent effect, with a PSS of ≥ 0.95. Additionally, without the addition of acid, the response intensity of the 10 mycotoxins was relatively low. The addition of FA enhanced the ion response intensity of the target compounds, with the average RRI rising from 25 ± 3 without FA to 87 ± 5 with FA. Furthermore, using methanol as the organic phase was more beneficial for separating target compounds from interfering impurities compared to using ACN. All 10 mycotoxins could be completely eluted within 10 min (Figure 2). The addition of FA also facilitated the ionization of different target compounds, ensuring good peak shapes and enhancing the response intensity of most target compounds. Consequently, water (0.1% FA)–MeOH (0.1% FA) was selected as the mobile phase.

### 3.4. Optimization of Mass Spectrometry Conditions

AFB1, AFB2, AFG1, AFG2, DON, FB1, FB2, and T-2 are readily ionized in positive ion mode, forming [M+H]+ quasimolecular ions, while OTA and ZEN form [M-H]- quasimolecular ions in negative ion mode. Therefore, the rapid positive–negative polarity switching function of tandem mass spectrometry is utilized to perform simultaneous scanning of samples in both positive and negative ion modes [24]. This approach allows for the acquisition of mass spectrometry information for all compounds with a single injection, thereby saving analysis time. Mass spectrometry conditions were optimized through single injection, scanning in both positive and negative ion modes (m/z 100–800) to select precursor ions. Based on the information of precursor ions, product ion scanning was conducted, and two precursor ion fragments were selected by comparing response values. The cone voltage was optimized using the signal intensity of the precursor ions, and the collision voltage was optimized using the signal intensity of the product ions. On the basis of the optimal mass spectrometry conditions, the MRM scan mode was established. For the same compound, the channel with a higher response value was used as the quantitative ion channel, and the channel with a slightly lower response value was used as the qualitative ion channel. Additionally, the source temperature, drying gas flow, and detector voltage of the mass spectrometer were optimized to ensure good response signals for all target compounds. The final precursor ions, product ions, and voltage parameters are shown in Table 1.

### 3.5. Method Validation

#### 3.5.1. ME Examination

ME primarily arises due to interference from co-eluting impurities in the sample matrix during the ionization process under ESI mode. This competitive ionization can either enhance or suppress the response of the target compounds, influencing the accuracy of quantitative analysis [25]. This interference is referred to as ME. The results showed that all 10 mycotoxins in Dendrobium officinale exhibited varying degrees of matrix suppression effects. The absolute MEs of T-2, FB1, and FB2 were within the range of 20.0%–50.0%, while those of AFG2, DON, and ZEN were within 10.0%–20.0%. The absolute MEs of the remaining mycotoxins were less than 10.0%, indicating that the MEs of most mycotoxins are relatively small. This allows for the use of internal standards and matrix-corrected standard curves to quantitatively remove MEs. Given the large number of mycotoxins and the difficulty and high cost of finding corresponding internal standards, the present study opted for matrix-corrected standard curves to achieve accurate quantification and eliminate the influence of MEs, thereby reducing detection costs. It is essential to note that while Dendrobium officinale is a critical representative of traditional Chinese medicinal plants, it is primarily the stem of this plant utilized for therapeutic purposes. The matrix properties of herbal medicines can vary significantly depending on the specific parts of the plant used, be it stems, leaves, or roots. For example, the chemical composition, physical properties, and potential for mycotoxin accumulation can differ between these parts, which may influence the extraction efficiency and subsequent detection of contaminants. Consequently, it is not appropriate to consider Dendrobium officinale as representative of all herbal medicines. Each type of herbal medicine may present unique challenges regarding mycotoxin contamination and should be studied within its specific context. By focusing our research on the specific matrices associated with Dendrobium officinale, we can provide a more accurate and relevant evaluation of mycotoxin levels that accounts for the plant's unique properties and usage.

#### 3.5.2. Linearity Range, LOD, and LOQ

The linearity range, correlation coefficient (R2), LOD, and LOQ for the 10 mycotoxins in Dendrobium officinale samples are shown in Table 2. The 10 mycotoxins exhibited good linearity within their respective linear ranges, with *R*^2^ > 0.990. The LODs and LOQs for the 10 mycotoxins in the blank matrix were 0.23–8.61 μg kg^−1^ and 0.77–28.7 μg kg^−1^, respectively, meeting the detection requirements of the national and industrial standards such as the National Food Safety Standard Limits of Mycotoxins in Food (GB 2761-2017) in China.

#### 3.5.3. Recovery and Relative Standard Deviation

As shown in Table 3, the average recoveries for the 10 mycotoxins in Dendrobium officinale samples ranged from 77.9% to 98.5%, with RSDs of 2.26%–8.28%. These results indicate that the method has high spiking recoveries and good repeatability, providing excellent accuracy and precision for the detection of all 10 mycotoxins in Dendrobium officinale. This meets the requirements of regulatory authorities regarding method precision and accuracy [26].

### 3.6. Detection of Actual Samples

The established method was utilized to determine 10 mycotoxins in 84 Dendrobium officinale samples from different regions. The results indicated that two samples tested positive for mycotoxins, with a detection rate of 2.38%. The primary contaminants in Dendrobium officinale were identified as AFB1, ZEN, and AFG1. One positive sample contained AFB1 and ZEN at concentrations of 2.8 ± 0.02 and 3.1 ± 0.03 μg kg^−1^, respectively, while the other positive samples contained AFB1, ZEN, and AFG1 at concentrations of 2.7 ± 0.02, 2.3 ± 0.01, and 3.2 ± 0.03 μg kg^−1^, respectively. These concentrations are consistent with findings in similar studies on herbal medicines. For instance, Caldeirao et al. reported AFB1 levels in various herbal extracts ranging from 74.3 to 1992.5 μg kg^−1^ [27], while Cho et al. noted ZEN levels in dried herbs from 2.88 to 15.21 ng g^−1^ [28]. This highlights the potential for mycotoxin contamination across different sources of Dendrobium officinale, suggesting that contamination can occur during various stages, including growth, harvesting, processing, storage, and distribution. The total mycotoxin concentrations in these two batches were 5.9 ± 0.06 and 8.2 ± 0.07 μg kg^−1^, respectively. Although these levels do not exceed the limit of 10 μg kg^−1^ total mycotoxins set for medicinal plants, as outlined in the National Food Safety Standards in China, they still pose health risks, particularly given the known toxicity of the detected mycotoxins. For example, AFB1 is classified as a Group 1 carcinogen by the International Agency for Research on Cancer (IARC), and even low concentrations can be hazardous with long-term exposure. The detection rate of 2.38% in this study is comparable to the contamination rates observed in other herbal products, which vary widely in the literature—from as low as 1% to as high as 10% in studies focused on herbal remedies [28]. These findings emphasize the importance of continuous monitoring for mycotoxin contamination in Dendrobium officinale and other herbal medicines to ensure safety and efficacy for consumers. The systematic analysis provided by this study offers critical data for regulatory bodies and quality control in the herbal medicine industry.

### 3.7. Comparison of Methodologies for Mycotoxin Detection

A variety of analytical methodologies have been developed for the determination of mycotoxins in herbal and edible plants. Among these, the QuEChERS extraction method combined with advanced chromatographic techniques, such as UHPLC–MS/MS, has gained popularity due to its efficiency and effectiveness. This is particularly advantageous for analyzing various mycotoxins in complex matrices like Dendrobium officinale, where interference from co-occurring compounds is common. Fontana et al. developed and optimized a QuEChERS LC–TQ–MS/MS to simultaneously determine the mycotoxins in complex medicinal plant matrices, and the LOQs of all four aflatoxins (B1, B2, G1, and G2) were 5 μg kg^−1^, and the LOQ of ochratoxin A was 10 μg kg^−1^ in *M. officinalis* [29]. Similarly, Huang et al. established a reliable and sensitive QuEChERS UHPLC–MS/MS method to simultaneously analyze 73 mycotoxins in edible and medicinal plants (lotus seed, coix seed, licorice root, and dried tangerine peel); method validation showed recovery rates of 61.6%–118.6% with RSDs below 15%, LODs of 0.25–12.25 μg kg^−1^, and LOQs of 0.5–25 μg kg^−1^ [30]. The QuEChERS method, as applied in this study, reduces processing time compared to traditional methods, enabling the analysis of multiple samples in a shorter period. The simplicity and reduced use of solvents and materials lower the overall cost of analysis, making it more accessible for routine screening. In conclusion, the QuEChERS–UPLC–MS/MS technique presented in this study not only stands out due to its robust performance and operational efficiency but also serves as a valuable method for ongoing research and regulatory monitoring for mycotoxin contamination in medicinal plants like Dendrobium officinale.

## 4. Conclusion

This study established a method for simultaneously determining 10 mycotoxins in Dendrobium officinale using QuEChERS and UHPLC–MS/MS through the optimization of sample purification conditions, sample pretreatment methods, chromatographic conditions, and mass spectrometric conditions. Compared to traditional methods using immunoaffinity columns, this approach significantly reduces detection costs. Samples were analyzed using ESI and MRM mode with matrix-matched external standard quantification. The 10 mycotoxin standard curves showed good linearity (*R*^2^ > 0.990), with LODs and LOQs of 0.23–8.61 μg kg^−1^ and 0.77–28.7 μg kg^−1^, respectively. The average recoveries at low, medium, and high concentrations ranged from 77.9% to 98.5%, with RSDs of 2.26%–8.28%. This method is user-friendly and provides excellent purification efficiency, high spiking recoveries, and reliable accuracy, meeting the requirements for large-scale rapid screening and accurate quantification of mycotoxins in Dendrobium officinale. It also provides technical reference for the simultaneous detection of multiple mycotoxins in other traditional Chinese medicine matrices. We applied this method to systematically test 84 Dendrobium officinale samples from different sources, discovering a 2.38% contamination rate of mycotoxins, primarily AFB1, ZEN, and AFG1. Although most medicinal plants maintain good sanitary standards during collection and processing, some samples still exhibit potential mycotoxin contamination risks. This study provides data support for investigating the contamination status of mycotoxins in Dendrobium officinale.

## Figures and Tables

**Table 1:** The optimal chromatographic and mass spectrometric parameters^∗∗^.

Compounds	Retention time	m/z		DP	CE
		Precursor ion	Product ion		
Aflatoxin B_1_	7.3	313.2	241.1^∗^; 284.9	90	50; 30
Aflatoxin B_2_	6.9	315.1	258.9^∗^; 287.0	110	40; 35
Aflatoxin G_1_	6.6	329.0	243.1^∗^; 214.2	110	40; 50
Aflatoxin G_2_	6.3	331.0	244.9^∗^; 189.0	110	45; 55
Deoxynivalenol	4.5	297.0	249.2^∗^; 231.1	60	15; 20
Fumonisin B_1_	8.1	722.3	352.2^∗^; 334.3	100	50; 50
Fumonisin B_2_	9.3	706.3	318.2^∗^; 336.4	140	40; 45
T-2	8.8	484.0	305.1^∗^; 185.2	40	20; 30
Ochratoxin A	9.5	404.2	166.9^∗^; 358.2	−80	−50; −25
Zearalenone	9.6	317.2	174.9^∗^; 131.0	−110	−30; −35

^∗^Transitions for quantitation.

^∗∗^Each parameter is based on single injection with multiple replicates for robust data consistency, ensuring reliable quantification of target compounds.

**Table 2:** The linearity and sensitivity of QuEChERS and UHPLC–MS/MS method for detecting mycotoxins in Dendrobium officinale.

Compounds^∗^	Linear equation	Linear range (μg·L^−1^)	*R* ^2^	LOD (μg·kg^−1^)	LOQ (μg·kg^−1^)
Aflatoxin B_1_	*y* = 1.625 × 10^4^*x* + 2.910 × 10^2^	0.5–50	0.9994	0.45	1.50
Aflatoxin B_2_	*y* = 3.721 × 10^3^*x* + 1.240 × 10^3^	0.25–25	0.9996	0.25	0.83
Aflatoxin G_1_	*y* = 3.567 × 10^3^*x* + 2.345 × 10^2^	0.5–50	0.9995	0.46	1.53
Aflatoxin G_2_	*y* = 4.892 × 10^3^*x* + 1.357 × 10^3^	0.25–25	0.9992	0.23	0.77
Deoxynivalenol	*y* = 2.123 × 10^3^*x* + 1.568 × 10^2^	10–100	0.9987	8.61	28.70
Fumonisin B_1_	*y* = 2.678 × 10^3^*x* + 1.123 × 10^3^	1–10	0.9991	0.82	2.73
Fumonisin B_2_	*y* = 1.234 × 10^4^*x* + 1.678 × 10^2^	1–10	0.9990	0.80	2.67
T-2	*y* = 3.567 × 10^3^*x* + 1.098 × 10^3^	1–10	0.9992	1.01	3.37
Ochratoxin A	*y* = 4.012 × 10^3^*x* + 1.456 × 10^3^	2–20	0.9997	1.42	4.73
Zearalenone	*y* = 3.987 × 10^3^*x* + 1.234 × 10^3^	2–20	0.9998	1.20	4.00

^∗^Each compound's linear range, LOD, and LOQ are shown, with *R*^2^ values indicating the method's predictive accuracy. Values are derived from multiple calibration curves based on three replicates for each concentration level.

**Table 3:** The recoveries of the mycotoxins in Dendrobium officinale at three different levels using QuEChERS and UHPLC–MS/MS method.

	Level (μg·kg^−1^)	Recovery (%)^∗^	RSD (%)		Level (μg·kg^−1^)	Recovery (%)^∗^	RSD (%)
AFB1	2.5	85.5	7.51	FB1	5	78.6	8.12
	5	85.7	5.48		10	80.1	3.66
	10	86.1	5.90		20	81.8	5.73

AFB2	1	87.4	3.58	FB2	5	77.9	8.28
	2	90.0	3.98		10	84.3	3.45
	4	88.6	4.16		20	80.0	6.27

AFG1	2.5	95.1	3.87	T-2	5	97.6	4.33
	5	96.3	2.26		10	98.5	3.89
	10	93.6	2.80		20	98.1	4.06

AFG2	1	89.7	4.12	OTA	5	85.7	5.29
	2	92.5	3.05		10	89.2	4.83
	4	90.6	2.98		20	86.4	5.01

DON	30	79.2	6.78	ZEN	5	79.2	7.12
	60	80.5	6.14		10	85.4	6.45
	120	79.8	5.45		20	83.9	5.55

^∗^Recovery percentages (%), along with relative standard deviations (RSD), were calculated from five parallel tests for each concentration level, demonstrating method robustness and accuracy across varying concentrations.

## Data Availability

The data that support the findings of this study are available from the corresponding author upon reasonable request.
